# KU-32, a Novel Drug for Diabetic Neuropathy, Is Safe for Human Islets and Improves *In Vitro* Insulin Secretion and Viability

**DOI:** 10.1155/2012/671673

**Published:** 2012-11-01

**Authors:** Kevin Farmer, S. Janette Williams, Lesya Novikova, Karthik Ramachandran, Sonia Rawal, Brian S. J. Blagg, Rick Dobrowsky, Lisa Stehno-Bittel

**Affiliations:** ^1^Department of Pharmacology and Toxicology, University of Kansas, 5064 Malott Hall, Lawrence, KS 66045, USA; ^2^Department of Physical Therapy and Rehabilitation Science, University of Kansas Medical Center, MS 2002, Kansas City, KS 66160, USA; ^3^Department of Medicinal Chemistry, University of Kansas, Lawrence, KS 66045, USA

## Abstract

KU-32 is a novel, novobiocin-based Hsp90 inhibitor that protects against neuronal glucotoxicity and reverses multiple clinical indices of diabetic peripheral neuropathy in a rodent model. However, any drug with potential for treating diabetic complications must also have no adverse effects on the function of pancreatic islets. Thus, the goal of the current study was to assess the effect of KU-32 on the *in vitro* viability and function of human islets. Treating human islets with KU-32 for 24 hours showed no toxicity as assessed using the alamarBlue assay. Confocal microscopy confirmed that with a minimum of 2-day exposure, KU-32 improved cellular viability by blocking apoptosis. Functionally, isolated human islets released more glucose-stimulated insulin when preincubated in KU-32. However, diabetic BKS-db/db mice, a model for type 2 diabetes, administered KU-32 for 10 weeks did not show any significant changes in blood glucose and insulin levels, despite having greater insulin staining/beta cell in the pancreas compared to untreated BKS db/db mice. In summary, KU-32 did not harm isolated human islets and may even be protective. However, the effect does not appear significant enough to alter the *in vivo* metabolic parameters of diabetic mice.

## 1. Introduction

Both type 1 and type 2 diabetes encompass elements of islet destruction, although by different mechanisms. Islet health is essential to maintaining normal glucose levels, and numerous approaches are being taken in attempts to prolong the life of the islet *β* cells. Previous studies have indicated that chaperone proteins, specifically heat shock protein 70 (Hsp70), can attenuate the cellular stress associated with type 2 diabetes and the eventual loss of islets [1]. 

KU-32 is a novel novobiocin-based Hsp90 inhibitor that can protect against neuronal cell death *in vitro* with minimal cytotoxicity to neurons [2, 3]. KU-32 protected against sensory neuron death and demyelination, and reversed the sensory deficits associated with diabetic peripheral neuropathy [4]. Mechanistically, the neuroprotective actions of KU-32 required the presence of a second molecular chaperone, Hsp70, because KU-32 was unable to reverse diabetic peripheral neuropathies in Hsp70.1 and Hsp70.3 double knockout mice [4]. 

Hsp70 has been identified within the insulin and amylin granules of pancreatic *β*-cells [5, 6], and its levels increased following exposure to interleukin-1*β* in cultured islets [7]. Overexpression of Hsp70 attenuated ER stress in cultured islets [8], and cellular death induced by NO donor compounds [7]. Conversely, while overexpression of Hsp70 has been associated with enhanced resistance to inflammatory mediators [9], actual heat exposure to isolated islets induced cellular apoptosis. Thus, activation of a cadre of heat shock proteins, including Hsp70, can cause apoptosis, resulting in increased graft failure after islet transplantation [10]. In fact, in pig islets, preconditioning with heat to activate Hsp70 protected the islet grafts from early inflammation after islet transplant, but enhanced eventual rejection of the islets by the recipient [11].

Recent data suggest that KU-32 may improve neuronal function by enhancing mitochondrial respiratory capacity following hyperglycemic stress [12, 13]. Since emerging research indicates that diabetes (especially type 2 diabetes) increases oxidative stress and mitochondrial fission/fusion in sensory neurons [14], the actions of KU-32 are enticing as a possible novel pharmacological intervention to reverse diabetic peripheral neuropathy. Given the complexity of Hsp70 on islet function and the critical role of this protein in KU-32-mediated neuroprotection, it is important to measure the effect of KU-32 on islet health and function since any therapeutic used to manage diabetic complications should, at the least, have a neutral if not beneficial effect on islets.

## 2. Materials and Methods

### 2.1. Human Islet Procurement

 Human tissues were obtained from the Integrated Islet Distribution Program (IIDP) and BetaPro (Gordonsville, VA). The samples of isolated islets were from 13 adult donors, whose characteristics are described in Table 1. None of the donors were diagnosed with diabetes or metabolic syndrome. Females comprised 46% of the sample donors, and the average age was 42 years. The majority of the donors were white with 23% African American, and no other donor minority status was identified. The average body mass index (BMI) was 29.55. Most donors died of head trauma (54%), 2 died of cerebral vascular strokes, and one of a gunshot wound. Three donors' cause of death was unknown. Isolated islets from the donors were maintained in CMRL 1066 medium with 2 mM glutamine, 10% FBS, and 1% antibiotic/antimycotic at 37°C in a culture chamber containing 5% CO_2_.

### 2.2. Animals

Male and female lepr mice (strain name: BKS.Cg-Dock7 m +/+ Lepr^ db ^/J) were purchased from Jackson Laboratory (Bar Harbor, ME, USA) and a breeding colony was established to generate animals with the homozygous genotype. Animals were housed 2 to 5 mice per cage and were kept on a 12 : 12-hour light/dark cycle with free access to food (5001 Purina Laboratory Rodent Diet, Purina Mills, St. Louis, MO, USA) and water. Weekly fasting blood glucose levels (One-Touch Ultra glucometer) were assessed and compared to age-matched heterozygous, nondiabetic litter mates. At 10 weeks of age, animals were given once per week intraperitoneal injection of 5% Captisol (CyDex Pharmaceuticals, Lenexa, KS, USA) or 20 mg/kg KU-32 in 5% Captisol. At termination of the study, blood from each animal was collected into 1.5 mL tubes containing 300 mM of EDTA, which was then centrifuged at 1500 ×g for 10 minutes at 4°C. The supernatant was collected and serum insulin levels were determined by ELISA (ALPCO, Salem, NH, USA, no. 80-INSMSU-E01). KU-32, [N-(7-((2R,3R,4S,5R)-3,4-dihydroxy-5-methoxy-6,6-dimethyl-tetrahydro-2H-pyran-2-yloxy)-8-methyl-2-oxo-2H-chromen-3-yl)acetamide], was synthesized and structural purity (>95%) verified as described in [15]. All animal procedures were performed in accordance with protocols approved by the University of Kansas Animal Care and Use Committee which is in compliance with National Institute of Health guidelines.

### 2.3. Toxicity Assays

 Possible toxicology of KU-32 was determined using alamarBlue (Invitrogen). Islets were placed into 96-well plates and subjected to a 8-point dose of KU-32 in either low (5 mM) or high (17.5 mM) glucose in DMEM : F12 media and incubated overnight at 37°C and 5% CO_2_. Twenty-four hours later, alamarBlue was added directly to each well to achieve a final concentration of 10% alamarBlue. Readings on a microplate reader (excitation wavelength = 530 nm and emission = 590 nm) were collected 4, 24, and 48 hours later. Blank wells containing media were used to determine background fluorescence. Data were calculated as Normalized Relative Fluorescence Units (NRFU/IE) according to manufacturer's instructions.

### 2.4. Viability Assay

 Islets were placed in a 500 mL volume of L-15 medium with live/dead fluorophores. The standard assay for cell apoptosis and necrosis included assessment of DNA fragmentation and loss of membrane integrity by exposing islets to YO-PRO-1 and propidium iodide (Vybrant Apoptosis Assay, Invitrogen, Grand Island, NY, USA, no. V13243) for 30 min at 37°C. Islets were rinsed with PBS and imaged with an Olympus Fluo laser scanning confocal microscope with a 20 or 40X objective. Background fluorescence was subtracted and viability percentages were calculated using FluoView software. The ratio representing the live cell area divided by the total islet area was calculated and reported as the percentage of dead cells. Islets were stressed, as indicated in the results section, by cycling high CO_2_ levels. Control and KU-32 treated islets were placed in stress media (DMEM with 1% FBS and 5.5 mM glucose). The islets were cycled between 5% CO_2_ and 20% CO_2_ for 2 hours at 37°C. 

### 2.5. Cellular Oxygen Consumption

Cellular oxygen consumption was completed using our published protocol [16]. Islets were exposed to 1 *μ*M KU-32 or vehicle for 24 hours in 96-well plates. The islets were transferred into oxygen-sensing, 96-well microplates (BD Biosciences), in Ham's media. Fluorescence of the individual wells, indicating relative changes in O_2_ consumption, was measured on a UV microplate reader (excitation wavelength of 485 nm, emission at 590–630 nm). The plate was read repeatedly over an 18-hour period. The values were normalized by subtracting the average of three blank wells from each measured well. Finally, values were divided by islet equivalencies to yield the O_2_ consumption/islet equivalency.

### 2.6. Protein Extraction and Western Blot

After washing, the islets were homogenized and protein extracted. Measurement of protein concentrations in supernatants was performed using BCA Protein Assay Kit (Pierce, Rockford, IL, USA, no. 23225). Proteins were separated on a 4–15% Tris-HCl Ready Gels (Bio-Rad Laboratories, Hercules, CA, USA, no. 161-1158) with 0.025 M Tris, 0.192 M Glycine, 0.1% SDS running buffer. Equal amounts of total protein (20 *μ*g) were loaded in each lane. After electrophoresis, the proteins were transferred from the gel to Bio Trace PVDF membranes 0.45 *μ*m (Pall Life Sciences, East Hills, NY, USA, no. P/N 66547), and visualized after staining in 0.1% Ponceau's solution, 5% acetic acid to ensure completeness of the transfer. Blots were blocked with 5% nonfat dry milk. Blots were probed with primary antibody against Hsp70 (ENZO Life Sciences, Plymouth Meeting, PA, USA, no. ADI-SPA-810) for 1 hour, dilution 1 : 1,000 followed by washing and exposure to secondary antibody horseradish peroxidase-conjugated goat anti-mouse IgG (Santa Cruz Biotechnology Inc., Santa Cruz, CA, USA, no. sc-2005), dilution 1 : 2,000. After repeated washings bound antibodies were detected using SuperSignal West Pico Chemiluminescent Substrate (Thermo Scientific Inc., Rockford, IL, USA, no. 34080). In order to normalize the protein loading by a housekeeping protein, the membranes were stripped, reprobed with antibody against actin (Sigma, Saint Louis, MO, USA, no. A2066, dilution 1 : 5,000) and processed with corresponding secondary antibody and the chemiluminescent substrate. Intensity of protein bands was quantified using Adobe Photoshop software. Protein loading was normalized by dividing intensity values for Hsp70-specific bands by intensity values for actin-specific bands. 

### 2.7. Insulin Secretion

 Static insulin secretion was measured from isolated human islets, placed in duplicate wells and assigned to two groups: low glucose (3 mM) and high glucose (16 mM) using our published protocol [17]. Briefly, islets in each well were exposed to KU-32 for varying times from 30 minutes to 24 hours. The media were changed and islets were exposed for 30 minutes in RPMI 1640 containing 3 mM glucose maintained at 37°C with 5% CO_2_. Low or high glucose solutions were added to the respective duplicates. After 30 minutes of static incubation at 37°C and 5% CO_2_, the islets were sedimented and the conditioned medium was collected and frozen at −80°C to determine insulin content by ELISA (ALPCO, Salem, NH, USA, no. 80-INSHU-E10.1).

Perifusion insulin secretion experiments were completed based on our published procedures [18]. Islets were incubated in CMRL 1066 overnight in a 37°C culture chamber containing 5% CO_2_ overnight, exposed to 1 *μ*M KU-32. Media were changed for a 2-hour preincubation. The islets were incubated in 5 mM RPMI 1640 with 10% FBS in 37°C incubator with 5% CO_2_ and placed in our custom perifusion chamber with a volume of 700 *μ*L, maintained at 37°C. Islets were initially perfused with 3 mM glucose RPMI 1640 with 10% FBS and 17 mM mannitol at a flow rate of 250 *μ*L per min for 30 minutes prior to sample collection. Samples were taken at 10-minute intervals for 60 minutes in low glucose. The glucose concentration was increased to 20 mM for 60 min, followed by a return to the 3 mM glucose concentration for 60 minutes. Collected fractions were flash frozen and stored at −80°C for determining insulin concentrations using a sandwich ELISA procedure (ALPCO, Salem, NH, USA, no. 80-INSHU-E01.1). 

### 2.8. Pancreatic Sections

The pancreata from mice were rapidly harvested and fixed in 10% normal buffered formalin. The tissues were subsequently dehydrated in graded concentrations of ethanol, cleared in xylene, and subsequently embedded in paraffin wax at 55°C. The tissues were sectioned at 8 *μ*m thickness, mounted on Superfrost/Plus microscope slides (Fisher Scientific, Pittsburg, PA, USA, no. 12-550-15) and dried at 40°C overnight and stored at 4°C until processing. The paraffin-embedded sections were deparaffinized/rehydrated in xylene followed by ethanol and PBS serial rehydration. Antigen retrieval was completed in 0.01 M citrate buffer, pH 6.2, with 0.002 M EDTA for 30 min using a steamer. Cells were permeabilized in 1.0% Triton X-100 in 0.1 M PBS, pH 7.4 for 30 min. Hematoxylin and eosin (H&E) staining was performed to illustrate the general islet morphology under light microscopy. 

### 2.9. Immunofluorescence Staining

 Sections were blocked in 10% normal donkey serum, 1.0% bovine serum albumin (BSA), and 0.03% Triton X-100 diluted in 0.1 M PBS, pH 7.4 for 30 min. Incubation with the primary antibody mix was performed at 4°C overnight in a wet chamber followed by incubation with the mix of fluorophore-conjugated secondary antibody at room temperature for 2 hr in a wet chamber protected from light. Both primary and secondary antibodies were diluted in 1% NDS, 1% BSA, and 0.03% Triton X-100. Slides were mounted with antifading agent Gel/Mount (Biomeda, Foster City, CA, USA, no. M01). The following primary antibodies were used: anti-insulin (1 : 100, Abcam, Cambridge, MA, USA, no. ab7842), antiglucagon (1 : 200, Abcam, no. ab10988), and antisomatostatin (1 : 200, Abcam, no. ab53165). Corresponding secondary antibodies were conjugated with Cy2 (1 : 200, Jackson ImmunoResearch Laboratories Inc., West Grove, PA, USA, no. 706-225-148), Alexa 555 (1 : 400, Molecular Probes, Eugene, OR, USA, no. A31570), and Alexa 647 (1 : 400, Molecular Probes, no. A31573). Images were collected using a Nikon C1si confocal microscope. 

### 2.10. Statistics

For insulin secretion assays, two-way ANOVA with Fisher's least significant difference test was used to compare groups. A *t*-test was used to compare total insulin content. Oxygen consumption data were analyzed using repeated measures ANOVA. For the immunostaining experiments, nested ANOVA was used. All figures include means ± SE. *P* value, defined as <0.05, was considered statistically significant.

## 3. Results

**Figure 1:**
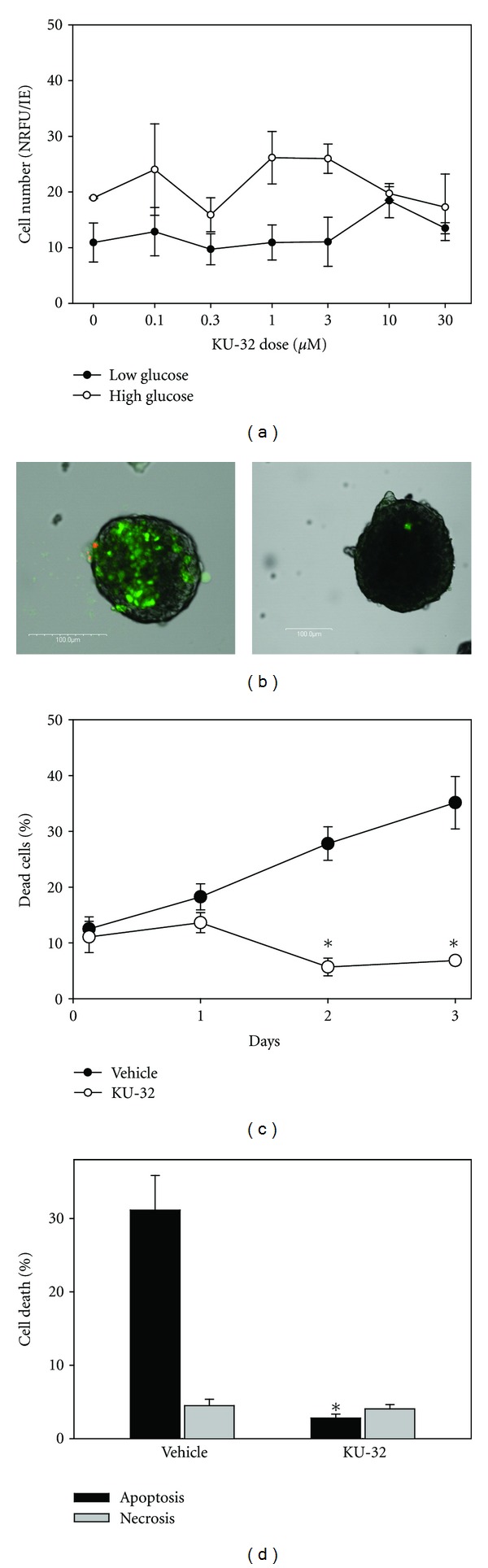
KU-32 improved viability of isolated human islets. (a) Toxicity studies using alamarBlue as an indicator of cell number shows no statistically significant toxic effect of KU-32 on human islets after a 24-hour exposure to 7 half-log doses (*n* = 6 replicates). (b) Representative islets are shown from vehicle-exposed islets (left) and KU-32- (1 *μ*M) treated islets. Viability was determined using fluorophores that indicate apoptosis (green staining) and necrosis (red staining). Scale bars = 100 *μ*m. (c) The percentage of cells within islets that were dead (stained red or green) were calculated as islets exposed to KU-32 or vehicle were maintained in cultures. (*indicates *P* < 0.001, *n* = 201 vehicle-treated and 177 KU-32-treated islets). (d) Further analysis of the percentage of cell death due to apoptosis or necrosis indicates that the majority of cell death was due to apoptosis in the vehicle islets while there was no difference in the low levels of apoptosis or necrosis in the KU-32-treated islets. (*indicates *P* < 0.001 between KU-32 and vehicle groups, *n* = 49 vehicle-treated and 59 KU-32-treated islets).

Isolated islets are extremely fragile *in vitro* due to cell death from both apoptosis and necrosis [16, 17]. If KU-32 were to be used as a clinical intervention for patients with diabetic peripheral neuropathy, any possible cytotoxicity to islets must be avoided. Initial cytotoxicity studies were performed in 5 mM glucose with doses of KU-32 from 0.03 to 30 *μ*M. After 24 hours of exposure to KU-32, there was no measureable cell loss to the islets exposed to any of the doses tested (Figure 1(a)). Replicate studies in high glucose also showed no signs of toxicity (results not shown). These initial toxicology screens, using the alamarBlue assay, provided general information concerning changes in cell numbers, but the natural heterogeneity in the size of native islets (ranging from 50–400 *μ*m diameters), along with the natural donor-to-donor variability, resulted in large variations, as noted by the error bars in Figure 1(a). Thus, we conducted additional viability studies using apoptosis/necrosis fluorophores and confocal microscopy. 

Figure 1(b) (left) provides a typical example of cell death associated with the vehicle-treated islets. The green staining indicates cell death due to apoptosis, and red is associated with necrotic cell death. In contrast, islets exposed to KU-32 showed less cell death (Figure 1(b), right). Islets maintained in culture have an increasing percentage of cell death, as we and others have shown [16, 17, 19, 20]. Exposure to KU-32 halted the time-dependent increase in cell death.  Figure 1(c) illustrates the progressive loss of viable cells/islets in the control group, while continued exposure to 1 *μ*M KU-32 for the duration of the experiment maintained cell death at around 10% in the treated group. Shorter time points were also tested starting with a 1-hour exposure. However, exposure durations of less than 48 hours failed to significantly alter cell viability. Of interest, the majority of cells died due to apoptosis.  Figure 1(d) shows that far fewer apoptotic cells were found in islets exposed to KU-32 compared to the controls. In contrast the amount of cell death due to necrosis was identical in both groups, but relatively low. 

**Figure 2:**
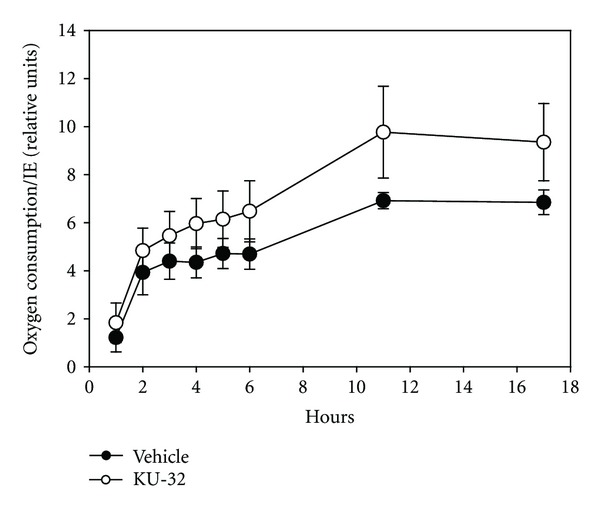
KU-32 increased oxygen consumption per islet. Exposure to KU-32 increased oxygen consumption per islet through an 18-hour period. Measurements were made in triplicate wells with 13–33 islet equivalents (IE)/well.

Further confirmation that KU-32 protected cells from apoptosis came from oxygen consumption studies. At each time point tested, oxygen consumption was greater per islet when exposed to KU-32 (Figure 2). Although the oxygen consumption per islet was increased with KU-32, when the oxygen consumption data were normalized to the percentage of viable cells within each islet (using the data shown in Figure 1), group differences in oxygen consumption were ablated (data not shown). In summary, the increased oxygen consumption associated with the KU-32 exposed islets was likely due to the increased number of live cells rather than changes in the cellular respiration.

**Figure 3:**
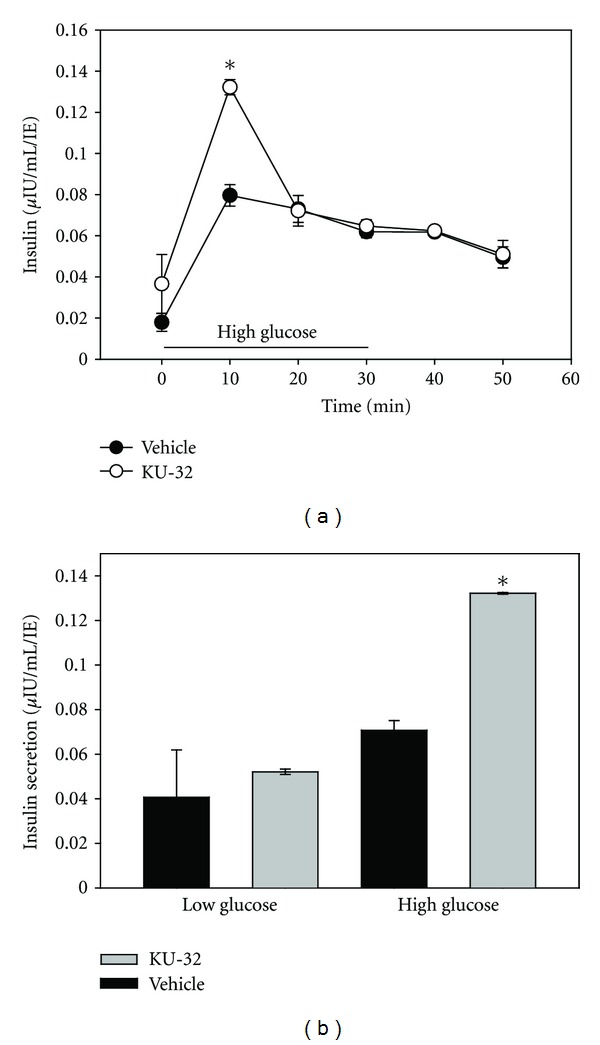
KU-32 increased insulin secretion. (a) In perifusion experiments, exposure to KU-32 increased insulin secretion in response to high-glucose. Initially, islets were maintained in 3 mM glucose. At time 0, the perfusate was switched to 17.3 mM glucose (high-glucose conditions). After 30 minutes, the glucose level was returned to 3 mM. During the high-glucose stimulation, there was significantly more insulin released by the KU-32-treated islets compared to the vehicle-treated. During low-glucose exposure, there was no difference between the 2 groups. (*indicates *P* < 0.05, *n* = 3 separate trials). (b) Insulin secretion measured during static incubation resulted in similar findings with statistically more insulin released in high glucose from the KU-32-treated islets. (*indicates *P* < 0.01).

To determine whether KU-32 altered function in *β* cells, perifusion experiments were performed on human islets. Islets were first exposed to low glucose (3 mM) for 90 minutes. Subsequently, the perfusate was switched to high glucose (20 mM) and the supernatant collected. While there was no significant effect by KU-32 on the insulin secreted in low-glucose conditions, there was significantly more insulin released in response to high glucose from the islets preexposed to KU-32 (Figure 3(a)). Repeated static incubation studies showed similar results with statistically higher insulin release in the KU-32 exposed islets (Figure 3(b)). In low glucose (3 mM), there was no effect of KU-32 on insulin secretion. The improved insulin secretion was demonstrated with a minimum of 16 hours of exposure prior to the assay. One-hour exposures to KU-32 failed to stimulate an improvement in insulin secretion even in high-glucose conditions (data not shown). 

**Figure 4:**
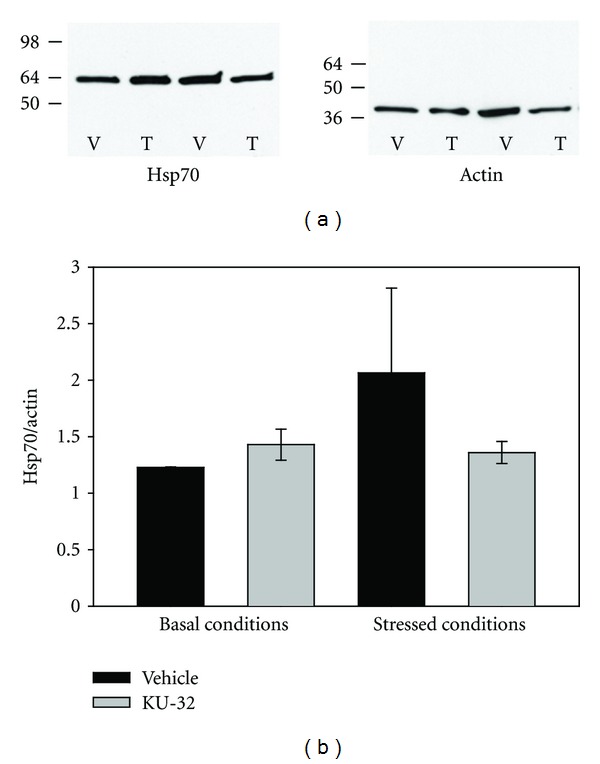
KU-32 failed to induce an increase in Hsp70 levels. (a) A typical western blot is shown with Hsp70 from samples of islets treated with vehicle (V) or KU-32 (T). MW size indicated on left. (b) Densitometry measurements of repeated western blots indicate that there was no difference in Hsp70 levels between vehicle- and KU-32 treated islet samples. When islets were stressed, Hsp70 levels increase and KU-32 exposure brought them back towards baseline, but these differences were not significant (*n* = 2 separate trials).

The molecular mechanism responsible for the neuroprotective effects of KU-32 depends on inhibition of Hsp90 and subsequent expression of Hsp70 [4]. Thus, we measured Hsp70 levels in human islets exposed to KU-32 for 24 hours.  Figure 4(a) presents a typical western blot recognizing Hsp70 and the housekeeping protein, actin. Surprisingly, there was no difference in Hsp70 levels between the vehicle- (V) and KU-32-treated (T) islets when normalized to actin levels (Figure 4(b)). Isolated islets normally express a high level of Hsp70; therefore, KU-32 may not have had an effect on quiescent islets. We stressed the islets by incubating them in hypoxic conditions with acidic media while exposed to either vehicle or KU-32. Control and KU-32 treated islets were placed in stress media (see methods) and cycled between 5 and 20% CO_2_. Under these conditions, islets had more Hsp70 in the stressed conditions (although not statistically different from basal conditions), but instead of KU-32 increasing the Hsp70 levels further, it returned Hsp70 back toward baseline levels (Figure 4(b)).

**Figure 5:**
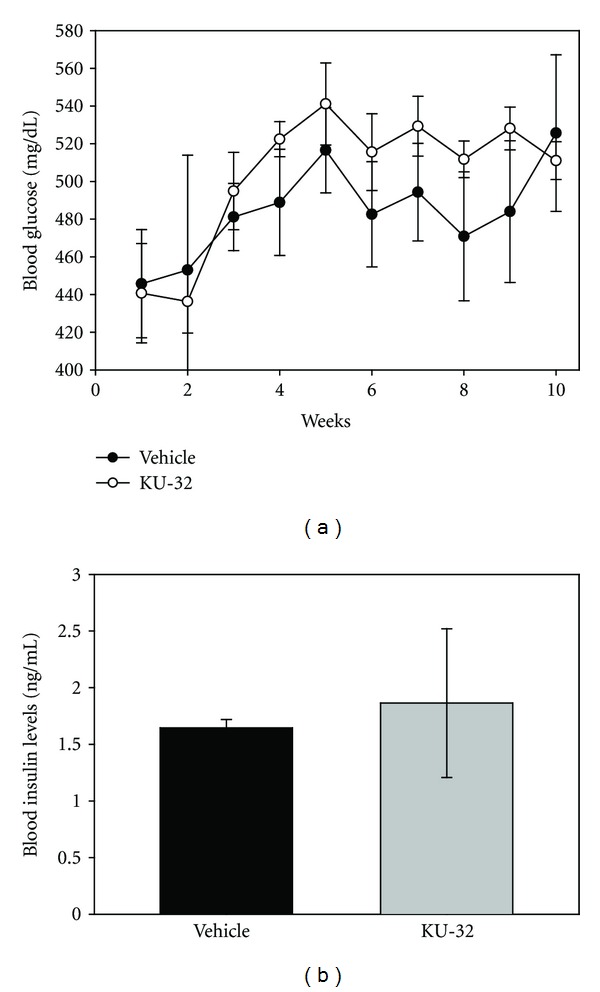
KU-32 did not alter blood glucose and insulin levels *in vivo*. (a) Weekly blood glucose measurements demonstrate that both groups of animals were diabetic, and levels were not affected by KU-32 administration (*n* = 12 vehicle- and 16 KU-32-treated mice). (b) At the termination of the study serum insulin levels were not different between the vehicle- and KU-32-treated mice (*n* = 3 vehicle- and 4 KU-32-treated mice).

In order to determine whether the protective action of KU-32 on islets existed *in vivo*, diabetic Lepr^ db^ mice were treated with weekly injections of KU-32. Elevations in blood glucose levels in these mice generally occur at ages 4 to 8 weeks of age. Therefore, once chronic hyperglycemia was confirmed, weekly KU-32 injections were administered starting at 10 weeks of age and continuing for 8 weeks. Blood glucose levels were not different between the vehicle and KU-32-treated animals (Figure 5(a)). Likewise, there was no difference in the serum insulin levels in the KU-32 versus vehicle-treated animals at the termination of the study (Figure 5(b)). Likewise, there were no signs of toxicity in the KU-32-treated animals.

**Figure 6:**
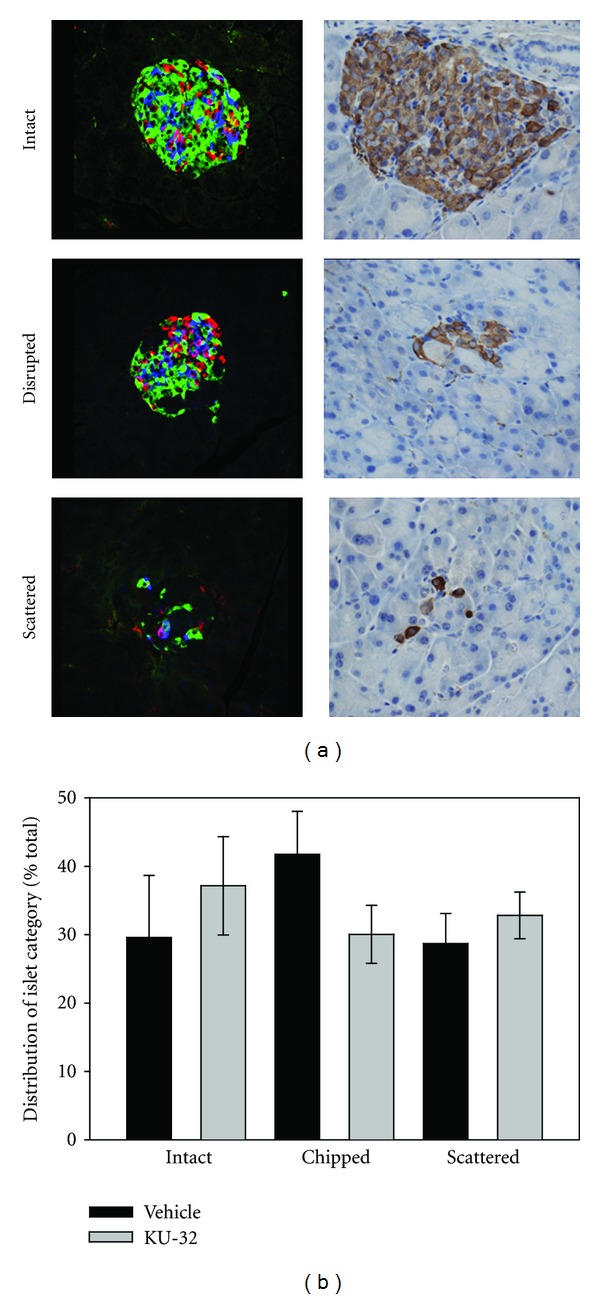
Lepr^ db^ mice had islets that were categorized into three types. (a) Immunofluorescence (left) and immunohistochemistry (right) illustrate the 3 types of islets that were identified in the Lepr^ db^ mice. Upper panels show intact islets, middle panels include disrupted islets, and lower panels show scattered islet cells. (b) There was no statistical difference in the distribution of the islet categories between the vehicle- and KU-32-treated mice pancreata (*n* = 12–16 sections from each of 3 mice/group).

At the termination of the study, the pancreata were removed and analyzed using immunofluorescence. Islet morphology was clearly different in the diabetic mice. Islets were either intact, or they had large areas devoid of *α*, *β*, or *δ* cells, termed disrupted islets [21]. Finally, there was a third category of islets that was composed of scattered endocrine cells, defining an area that had once been an islet. These morphological categories could be viewed with both immunofluorescence and immunohistochemistry techniques (Figure 6(a)). Both vehicle- and KU-32-treated animals had islets that fell into the three categories and there was no difference in the distribution of islets within each category between groups (Figure 6(b)).

**Figure 7:**
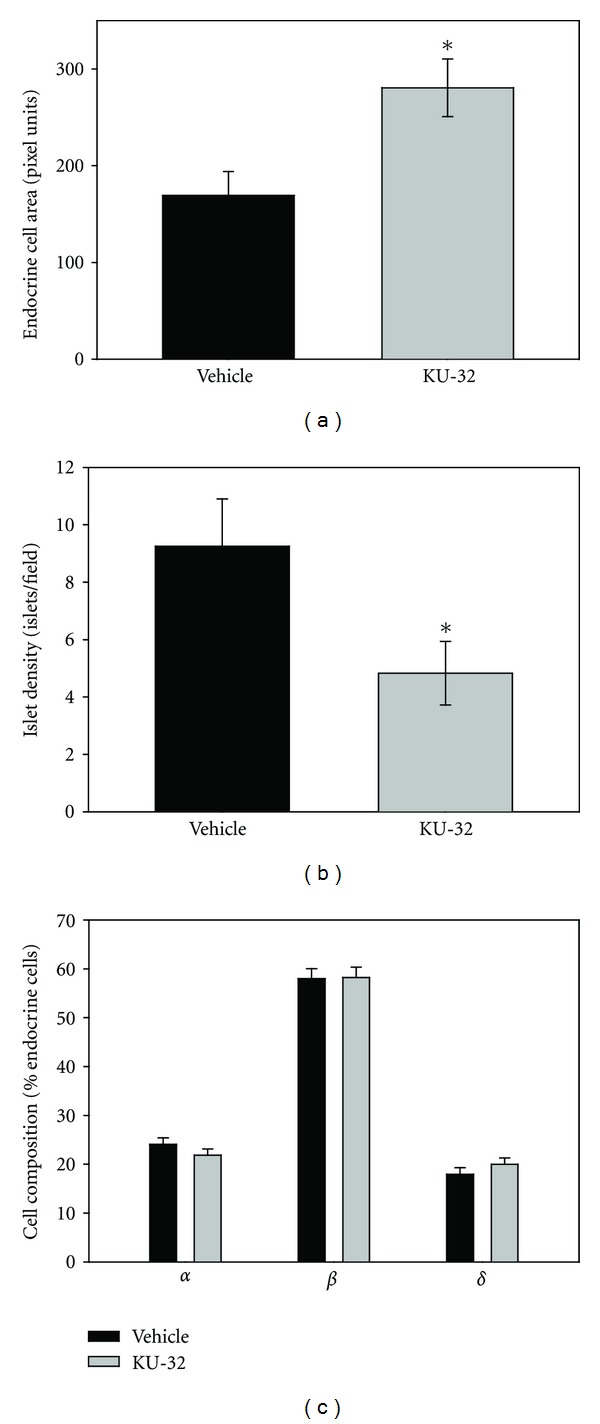
KU-32 improved the total islet area, but not the islet density or cell composition. (a) Treatment with KU-32 increased the total area of the pancreas identified by immunohistochemistry as endocrine cells. (*indicates *P* < 0.007, *n* = 40 islet from vehicle-treated mice, and 46 islets from KU-32 treated mice). (b) KU-32 decreased the density of islets per pancreatic section analyzed. (*indicates *P* < 0.05, *n* = analysis of 4 sections from 3 vehicle-treated mice and 6 sections from 3 KU-32-treated mice). (c) There was no difference in the percentage of *α*, *β*, or *δ* cells in each islet analyzed. (*n* = 56 islets from vehicle-treated mice and 40 islets from KU-32 treated mice).

From the serial sections, islet area was measured, and there was a statistically significant increase in total area of the endocrine-stained cells in the KU-32-treated mice (Figure 7(a)). In summary, more total pancreatic area was composed of *α*, *β*, or *δ* cells with KU-32 treatment. However, the density of islets per microscopic field in the pancreas tail decreased in the KU-32-treated animals (Figure 7(b)). 

We hypothesized that KU-32 would spare some of the diabetes-induced loss of endocrine cells within the islets, by sparing the *β*-cells specifically. Thus, we compared the percentage of *α*, *β*, and *δ* cells within the islets. These measurements were only made on intact and disrupted islets (not scattered cells).  Figure 7(c) shows that there was no difference between the two groups, with both groups of islets showing a dominant percentage of *β*-cells (Figure 7(c)). 

**Figure 8:**
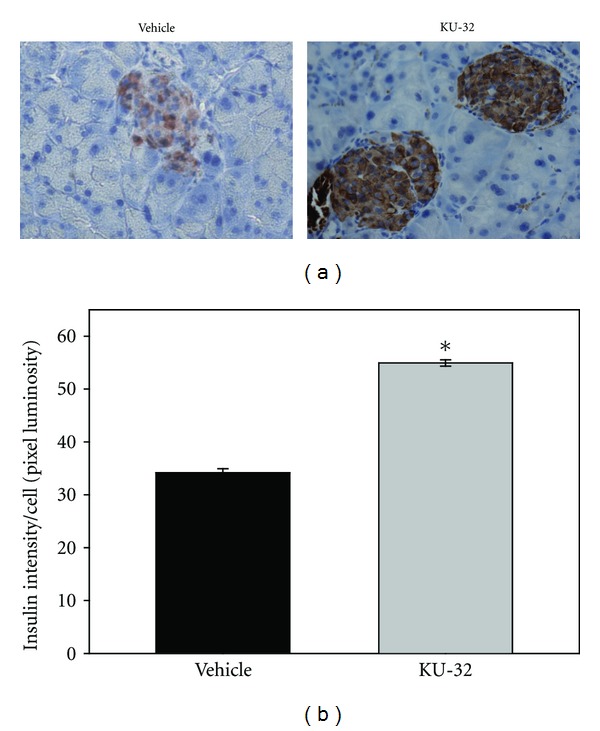
Insulin staining intensity per cell was higher with KU-32 treatment. Representative examples are shown of insulin immunohistochemistry for islets from vehicle-treated and KU-32 administered mice. (b) Insulin staining intensity values (brown) were background-subtracted (blue) and calculated as pixel intensity. KU-32 induced a significant increase in insulin staining. (*indicates *P* < 0.001, *n* = 313  *β* cells from 7 vehicle-treated mice and 682 *β* cells from 5 KU-32-treated mice).

While there was no difference in the percentage of *β*-cell to other endocrine cells with KU-32 administration, there was a clear difference in the intensity of the insulin staining, which is indicative of the insulin level/cell. Insulin staining intensity was greatest in the KU-32-treated animals when calculated per islet (Figure 8(a)) and per *β*-cell (Figure 8(b)). 

## 4. Discussion

Approximately 26 million Americans are diagnosed with diabetes and approximately 60–70% of those individuals develop diabetic peripheral neuropathies. To date, there are no effective treatment options specific to the neuropathy. KU-32 is a small molecule designed to inhibit Hsp90, thereby increasing Hsp70 levels. Both Hsp70 and Hsp90 are molecular chaperones that are critical for the proper folding of proteins. KU-32 has been shown to have dramatic effects in protecting neurons from stress and even reversing the clinical signs of diabetic neuropathy in animal models. Treatment of diabetic rodents reversed multiple clinical indicators of diabetic peripheral neuropathy including thermal hypoalgesia [4] and mechanical sensitivity [12]. If it is to be administered as an antineuropathy drug to diabetic patients, any possible effects on islets must first be identified. 

In this study, we showed that KU-32 improved human islet cell viability and long-term survival in culture with no signs of toxicity. In fact, over a 3-day period there was 25% less cell death in cultured human islets incubated with KU-32 compared to those exposed to vehicle. It was interesting that the KU-32-induced decrease in cell death was due to fewer apoptotic cells, while there was no change in the amount of necrotic core cell death with KU-32 exposure. 

Glucose-stimulated insulin secretion was increased in human islets exposed to KU-32 for 24 hours, when measured with static incubation or perifusion methods. It is important to note in both situations that KU-32 had no effect on insulin secretion in low-glucose concentration. Clinically, if the compound induced an increase in insulin under low-glucose conditions, any animal or person exposed to that drug would be at risk for hypoglycemic events, as glucose-sensitive insulin secretion is key to normal regulation of blood glucose levels.

Interestingly, the *in vitro* effects of KU-32 did not appear to use the same cellular mechanism in islets as neurons. In rodent nerves, KU-32 increased Hsp70, and this protein was necessary for the neuroprotective efficacy of the drug in reversing indices of diabetic peripheral neuropathy [4]. In contrast to the rodent neurons, KU-32 did not induce an increase in Hsp70 in isolated cadaveric human islets. However, it has been shown that human islets express significantly higher levels of Hsp70 than rodents even under quiescent conditions, and human islets have a dramatically smaller dynamic response to heat and stress compared to rodent Hsp70 levels [22, 23]. Our results support this conclusion, as Hsp70 levels in rat muscle (used in the western blots as a positive control) were nearly 1000 times less than human islets levels (data not shown). New data suggest that other molecular mechanisms may be activated in response to KU-32, such as mortalin/Grp75, Hsp60 and antioxidant proteins such as Mn superoxide dismutase [13]. Thus, the lack of increase in Hsp70 levels in human islets in our study should not be surprising. 

*In vivo* KU-32 did not reverse the blood glucose levels of severely diabetic mice. This may have been because the islet integrity was too diminished before the administration of KU-32 was initiated. While KU-32 did not spare islets from the degradation associated with diabetes, it did maintain a statistically higher level of insulin per *β*-cell. It would be interesting to determine whether a greater effect of KU-32 could be measured, if the administration of the compound was initiated earlier in the disease process. In fact, at the initiation of KU-32 administration in this study, the blood glucose levels were approximately 450 mg/dL, and it is likely that the mice were already hyperinsulinemic, although we did not measure blood insulin levels. Future studies will examine the *in vivo* effect of KU-32 when administered earlier in the course of the disease.

## 5. Conclusions

KU-32 is a small molecule designed to reverse diabetic peripheral neuropathy, which it does effectively in rodent models of diabetes. In cadaveric human islets, it enhanced *in vitro* insulin secretion and improved islet viability in culture. Yet, it did not improve glucose control in the db/db mice at the time point tested. Certainly, KU-32 has been shown in this study to be nontoxic to human islets at clinically relevant doses and may be beneficial to islet health *in vitro*. The *in vitro* implications of this compound should not be overlooked. Laboratories have worked feverishly to add molecules to islet media that would enhance their *in vitro* survival, both for research purposes and as they prepare and ship islets for transplantation into people with diabetes. KU-32, or related analogs [24], may prove to be a useful compound towards this goal as well as effectively treating diabetic neuropathy.

## Figures and Tables

**Table 1:** Characteristics of human islet donors.

Sex	Age (years)	BMI	Race	Cause of death
F	44	22.5	White	Head trauma
M	30	27.6	White	Head trauma
M	30	26.2	White	Cerebrovascular stroke
M	44	30.4	White	Head trauma
F	55	26.8	AA	Head trauma
F	47	26.3	White	Cerebrovascular stroke
F	58	25.8	Unknown	Unknown
F	38	30	AA	Unknown
M	54	32	White	Gunshot
F	26	33	AA	Head trauma
M	44	26.3	White	Head trauma
M	49	34.5	Unknown	Unknown
M	27	33	White	Head trauma

BMI: body mass index; M; male; F: female; AA: African American.
